# Evaluation of Synergic Potential of rGO/SiO_2_ as Hybrid Filler for BisGMA/TEGDMA Dental Composites

**DOI:** 10.3390/polym12123025

**Published:** 2020-12-17

**Authors:** Ali Alrahlah, Rawaiz Khan, Abdel-Basit Al-Odayni, Waseem Sharaf Saeed, Leonel S. Bautista, Fahim Vohra

**Affiliations:** 1 Restorative Dental Sciences Department, College of Dentistry, King Saud University, Riyadh 11545, Saudi Arabia; 2 Engineer Abdullah Bugshan Research Chair for Dental and Oral Rehabilitation, College of Dentistry, King Saud University, Riyadh 11545, Saudi Arabia; aalodayni@ksu.edu.sa (A.-B.A.-O.); wsaeed@ksu.edu.sa (W.S.S.); lbautista@ksu.edu.sa (L.S.B.); fvohra@ksu.edu.sa (F.V.); 3 Department of Prosthetic Dental Science, College of Dentistry, King Saud University, Riyadh 11545, Saudi Arabia

**Keywords:** hybrid filler, dental composites, mechanical properties, viscosity, degree of conversion

## Abstract

Graphene and graphene oxide based nanomaterials have attained immense significance in research because of their matchless physiochemical characteristics. Although potential biomedical applications of graphene have been extensively studied, however, dentistry related applications were rarely explored. This study aimed to investigate the effect of various percentages of surface modified reduce graphene oxide (S-rGO) in combination with SiO_2_ nanoparticles (bulk filler) on numerous physio-mechanical characteristics of acrylate-based (BisGMA/TEGDMA: 1:1 by wt.) composites. BisGMA/TEGDMA reinforced with 30 wt.% surface modified fumed-silica (S-A200) was considered as control group (base composite). Various concentrations (0, 0.5, 1, 2, 4 wt.%) of S-rGO were incorporated into the base composite via solution casting and high-speed mixing. The obtained composites were characterized for rheological properties before curing by using Rheometer (Anton Paar, USA) in the oscillatory mode under a frequency sweep over a range of angular frequency of 0.1–100 rad/s at 25 °C. The degree of conversion (DC) was measured by using Fourier transform infrared spectroscopy (FTIR). A Nano-indentation test was carried out to obtain nano-hardness and elastic modulus. The surface roughness was measured by optical microscope (Bruker^®^), 3D non-contact surface profilometer. The structural and morphological properties were studied by using Scanning Electron Microscopy (SEM). The mean and standard deviation were calculated and a simple mean comparisons test was performed for comparison using SPSS. The results revealed that the addition of a tiny proportion of S-rGO considerably increased the nano-indentation hardness, elastic modulus and DC. Conversely, a gradual reduction in viscosity was observed with increasing S-rGO concentration. The study demonstrates that a small fraction of S-rGO in combination with SiO_2_ could enhance physical, mechanical and rheological properties of acrylate based composites. Thus S-rGO/SiO_2_ combination could be used as a potential hybrid filler for dental nanocomposites.

## 1. Introduction

Bisphenol A glycerolate dimethacrylate (BisGMA) has been commonly used as a significant dental base monomer since early 1960s, owing to its suitable mechanical properties, fast restoration with the benefit of low volumetric shrinkage upon polymerization and higher resin reactivity [1,2]. However, its high viscosity causes low DC of the monomer [3]. Therefore, triethyleneglycol dimethacrylate (TEGDMA) is usually added as diluent to BisGMA in order to enhance its handling characteristics [4]. In the BisGMA/TEGDMA resin systems, BisGMA causes a reduction in volumetric shrinkage persuaded by photo-polymerization and increase resin reactivity while TEGDMA improves vinyl double-bond conversion [5,6]. Camphorquinone (CQ) is mostly used as a photoinitiator in conjunction with electron donor tertiary amine: Dimethyl amino ethyl methacrylate (DMAEMA) and Ethyl-4-dimethyl aminobenzoate (EDMAB) as co-initiator. The reason for preferably using CQ as a photo-initiator is its broad absorbance range (360–510 nm) and peak absorbance at 468 nm in visible light spectra [7]. Although BisGMA/TEGDMA resin systems are widely used in dental restorative materials. However, obtaining adequate mechanical and physical properties is still an ongoing topic of research [8].

Graphene is a two-dimensional new allotrope of carbon, one atom thick planer sheet comprised of sp2 hybridized carbon atoms organized in a crystal honeycomb lattice. It is considered not only the thinnest known material [9,10] but also the strongest and stiffest material available to-date with Young’s modulus of around ~1 TPa and strength of ~130 GPa [11]. It has many outstanding physicochemical characteristics like enormously large surface area, good optical transmittance (≈98%), high thermal and electrical conductivity and high mechanical properties [10], which make it a favorable nano-filler for polymer nanocomposite. Reduced graphene oxide (rGO) is an intriguing derivative of graphene and is the best suitable choice for large scale application due to its ease of production as compared with perfect single-layer or few-layer graphene. Similarly, SiO_2_ is a multi-purpose nanofiller used to fabricate multifunctional polymer nanocomposites and due to its amorphous nature and high specific surface area (≥450 m^2^/g), it has good miscibility with polymer matrix. Its high hardness and lower price compared to other fillers make it an ideal filler [12]. However, the particles tendency towards agglomeration restricts its concentration in the matrix [13,14].

The polymer/graphene based nanocomposite has been an interesting research area for many years and various polymers have been intermingled with graphene [15,16,17,18,19]. The incorporation of graphene has greatly improved the mechanical properties of the polymers. Previous studies revealed that the tensile strength, Young’s modulus and Tg (glass transition temperature) of Poly(methyl methacrylate) (PMMA)/graphene nanocomposites has been magnificently enhanced by the incorporation of grapheme [20,21]. Besides, new class of nanocomposites based on graphene and its derivatives has been introduced by using them as fillers for polymeric materials [22,23,24]. The physical and mechanical properties of the polymers were tremendously improved upon the incorporation of a small proportion of graphene [23,25]. In the literature, GO-SiO_2_ hybrids have been incorporated as filler into epoxy polymer matrix and demonstrated remarkable improvement in properties compared with pristine GO nano-sheets and SiO_2_ nanoparticles individually [26,27]. Similarly, Haeri et al. [28] incorporated graphene oxide nanosheets functionalized with silica nanoparticles as filler into epoxy resin. They reported significant improvement in the mechanical properties by the inclusion of GO-SiO_2_ nanoparticles. Remarkable improvements in mechanical properties were reported by Ozcan et al. [27] by using Silica/Graphene nanohybrid particles. The addition of even a small amount of graphene into BisGMA/TEGDA) polymer matrix turned out significant improvement in modulus of elasticity and surface hardness [29]. GO-SiO_2_ hybrids demonstrated tremendous improvement in the physical and mechanical properties of epoxy resin. Various other combinations of hybrids have been used by researchers to improve the mechanical and physical properties by bringing hybrid filling technology and novel reinforcing fillers [30,31]. 

Since the combination of Graphene/SiO_2_ based hybrid fillers demonstrated tremendous improvement in the physical and mechanical properties of other polymers. The current study aimed to discover the synergic potential of rGO/silica as a hybrid filler for dental composites. For this purpose, various concentrations of S-rGO (0.5, 1, 2, 4 wt.%) in combination with a bulk proportion of surface-modified fumed-silica (30 wt.% silane modified as dispersed phase) as hybrid filler were dispersed in BisGMA/TEGDMA resin (resin/filler ratio as 70/30) and evaluate its effect on viscosity, degree of conversion, mechanical properties (nano-hardness, elastic modulus) and morphology of nanocomposites. The study intended to discover the synergic potential of rGO/silica as a hybrid filler for dental composites. 

## 2. Material and Methods

### 2.1. Materials 

Bisphenol A glycidyl methacrylate (BisGMA; 98%), Triethylene glycol dimethacrylate (TEGDMA; >95%), Camphorquinone (CQ; 97%), 2-(Dimethylamino) ethyl methacrylate (DMAEMA; 98%), 3-(Trimethoxysilyl) propyl methacrylate (γ-MPS; 98%) and reduced graphene oxide powder (rGO) (carbon > 65 wt.%, nitrogen > 5 wt.%) were purchased from Sigma-Aldrich, Taufkirchen, Germany. Ethanol absolute (EtOH, >99.8%) and acetic acid (AcOH, >99%) were supplied by Alfa Aesar, Karlsruhe, Germany. Amorphous fumed silica (Aerosil^®^ 200, A200) (12 nm particle size, 200 m^2^/g BET surface area, 1.5 mass loss on drying and 3.7–4.5 pH) was obtained from Evonik-Degussa, Essen, Germany.

### 2.2. Modification of Fillers 

The fillers used in this investigation was silanated using the organosilane, γ-MPS, prior to application. Aerosil^®^ 200 (A200) was silanated using 10 wt.% of γ-MPS (enough to completely cover the surface of the silica and to attain tenacious interphase) following a method reported elsewhere [32,33,34]. The minimum amount of silane (*X*%) required for filler particles coverage can be calculated using Equation (1) [32,35], in which *A* is the surface area of the filler (A200, 200 m^2^/g) and *w* is the surface area coverage per g of silane which is 2525 m^2^/g for γ-MPS [35,36].

(1) $$X\left(\%\right)=\frac{A}{w}\;\times 100.$$

The γ-MPS was prehydrolyzed for 2 h in 70 wt.% ethanolic aqueous solution to which a few drops of acetic acid were added to adjust the pH value approximately to 3–4. The A200 was added to the solution and stirred at 60 °C for 30 min, then equilibrated with stirring at room temperature for 24 h. The slurry was sonicated for 10 min, filtered and washed three times with ethanol. The obtained silanized silica (S-A200) was re-suspended in ethanol, sonicated for 10 min and finally allowed to dry in a vacuum oven overnight at room temperature. 

Modification of rGO with the coupling agent γ-MPS was attempted using a method modified from literature [32,33,37]. In this case, γ-MPS (0.05 mL) was separately prehydrolyzed in 15 mL ethanolic aqueous solution (70 wt.%) for 2 h. The silane solution was added dropwise into a pre-suspended rGO (400 mg) in 250 mL of 70 wt.% ethanolic solution and refluxed at 60 °C for 2 h, then kept on stirring overnight at room temperature. Finally, the silanized rGO (S-rGO) was collected by centrifugation and washed several times with 70 wt.% ethanol, sonicated in 5 mL ethanol and dried as above. 

### 2.3. Preparation of Dental Composites

**Figure 1:**
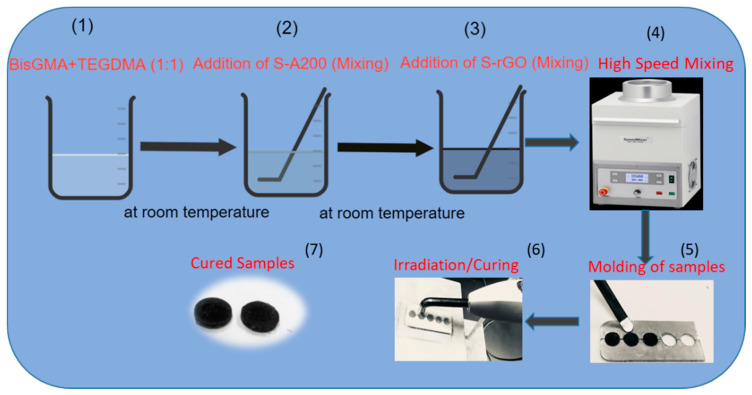
Schematic of BisGMA/TEGDMA (bisphenol A glycidyl methacrylate/ triethylene glycol dimethacrylate) dental composites filled with S-A200 and S-rGO.

A series of dental composites were prepared as per the schematic given in Figure 1. Each composite consists of 70 wt.% resin (1:1 BisGMA/TEGDMA) and 30 wt.% silanated-filler, in which the amount of S-A200 was sequentially replaced with 0.0, 0.15, 0.30, 0.60 and 1.20 wt.% of S-rGO (representing 0, 0.5, 1.0, 2.0 and 4.0 wt.% with respect to the total fillers); denoted C0.0–C4.0 as given in Table 1. Typically, the initiator system was dissolved in the pre-prepared resin mixture. The defined fillers of each composite were manually homogenized and hand-mixed, in batches, with the respective amount of the resin using a stainless steel spatula. The components were further mechanically mixed using a dual asymmetric centrifuge at 2500 rpm, three times with two min rest in-between. The fabricated composites were kept in dark containers at 8 °C until use.

## 3. Characterization

Fourier Transform infrared (FTIR) spectra of the fillers were recorded on a Nicolet iS10 spectrometer (thermo scientific, Madison, WI, USA) using a KBr-disc method over the range of 4000–400 cm^−1^, resolution of 4 cm^−1^ and scanning cycles of 32 per spectrum. The degree of conversion of the model composites was determined under the same measuring-setup using an attenuated total reflection accessory (ATR; diamond crystal) equipped with the instrument. The rheological properties of the composites were measured using MCR-72 rheometer (Anton Paar, Graz, Austria) at the oscillatory mode under a frequency sweep pre-set condition of 8 mm parallel plate, 0.25 mm gap and over a range of angular frequency of 0.1–100 rad/s at 25 °C. The morphology and structure of all the obtained composites were studied by scanning electron microscopy (SEM) (JEOL, JSM-6610 LV, Tokyo, Japan) at 15 kv voltage and resolution of 1000. 

### 3.1. Nano-Indentation, Surface Roughness and Morphology

**Figure 2:**
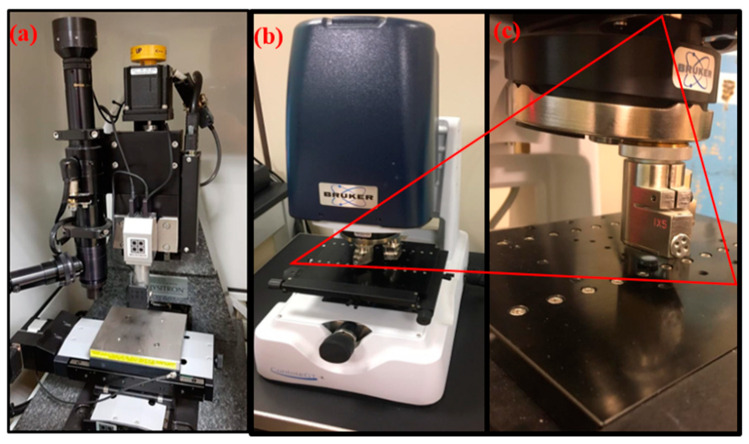
(**a**) Hysitron Nano-mechanical testing instrument, (**b**) optical microscope (Bruker^®^), 3D non-contact surface profilometer, (**c**) magnified image of mounted sample on stage.

Nanoindentation measurement was conducted using Hysitron (TI 700 Ubi 1) nano-mechanical testing instrument, (Bruker®, Billerica, Massachusetts, USA) (Figure 2). This instrument is equipped with a pyramidal (Berkovich 142.3 degree, three sided diamond probe) indenter. Moreover, an electronically adjustable camera (10× Zoom) is installed to focus and analyze the areas of interest in the sample. The nano-indenter monitors and records the applied load (F) and displacement (d) of the indenter. Tests were performed at a room temperature of 25 ± 0.5 °C. The system calculates the absolute nano-hardness and reduced modulus (Er). The surface roughness was measured using a contour GT-K 3D optical microscope (Bruker^®^), 3D non-contact surface metrology with interferometry. Samples were measured by vertical scan interferometry using 5x Michelson magnification lens with a field of view of 1.0 × 1.0 mm^2^, Gaussian Regression Filter, a scan speed of 1x and thresholding of 4.

Samples were placed on the stage and manually adjusted to give an image on the monitor screen. The microscope uses a Vision 64 (Bruker^®^) software which controls the instrument settings, data analyses and graphical output. The measurement was performed using vertical scanning interferometry which uses a broadband (normally white) light source which is effective for measuring objects with rough surfaces, as well as those with adjacent pixel-height differences greater than 135 nm. Each sample was scanned at 3 selected positions with 3 intervals and averaged accordingly to determine roughness (Ra) value. The mean and standard deviation were calculated and a simple mean comparisons test was performed for comparison using SPSS.

### 3.2. Degree of Conversion (DC)

The degree of double bond conversion was determined using FTIR method and all samples were treated similarly following a typical protocol [38]. Thus, the specimen to be measured was sandwiched between two glass slides in a fabricated disc-shaped plastic mold (5 × 2 mm^2^, n = 5) and irradiated for 60 s using a 3M curing unit (3M ESPE-S10, LED curing light, wavelength of 430–480 nm, intensity of about 1200 mW/cm^2^). Using the fact that the ratio between peak areas of the same type of chemical bonds, in the same spectrum, could reflect their real mole ratio as well, the peak areas of the aliphatic (at 1637 cm^−1^, polymerizable) and aromatic (1608 cm^−1^, un-polymerizable) C=C, before and after curing, were compared; thus, the *DC* was calculated using Equation (2). The mean and standard deviation were calculated and a simple mean comparisons test was performed for comparison using SPSS.

(2) $$DC\;\left(\%\right)=\left[1-\frac{{\left(\frac{A_{1637}}{A_{1608}}\right)}_{cured}}{{\left(\frac{A_{1637}}{A_{1608}}\right)}_{uncured}}\right]\;\times \;100.$$

## 4. Result and Discussions

### 4.1. Surface Modification of Fillers

**Figure 3:**
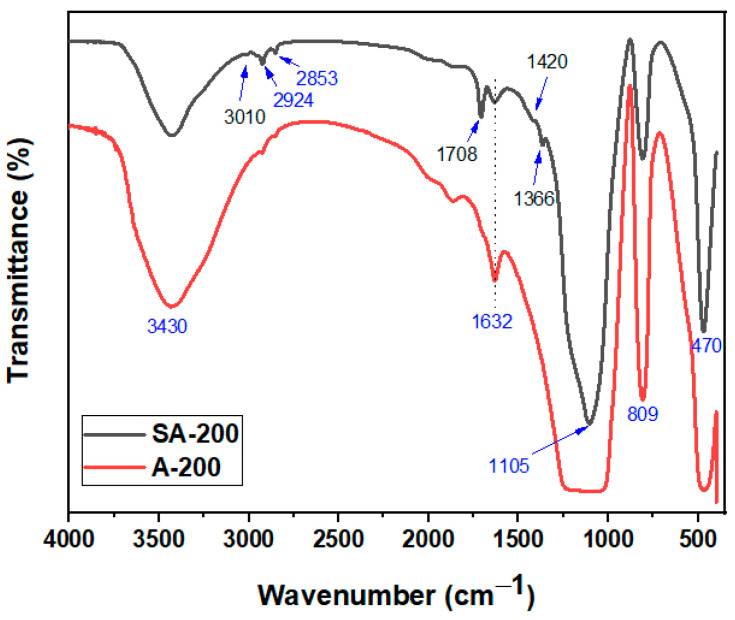
Fourier transform infrared (FTIR) spectra of fumed silica before (A200) and after modification (S-A200).

Surface modification of silica (A200) was confirmed via Fourier transform infrared (FTIR) spectroscopy (Figure 3). The characteristic peaks corresponding to the hydrophilic silica (A200) were observed at 3430 cm^−1^ for OH stretching, at 1632 cm^−1^ for silanol OH bending as well as for water twisting band, at 1105 and 809 cm-1 for asymmetric and symmetric stretching mods of siloxane (Si–O–Si) framework and at 470 cm^−1^ for Si–O rocking vibrations [32,33,39]. The spectrum of the silanized silica, S-A200, revealed additional peaks at 3010 cm^−1^ for =C–H (carbon-sp^2^) stretching, 2924 and 2853 for C–H (carbon-sp^3^), 1708 for C=O stretching, 1420 for C=C stretching and at 1366 cm^−1^ for C–H bending mode all assigned to the γ-MPS molecules, thus indicating successful modification of A200 silica. 

**Figure 4:**
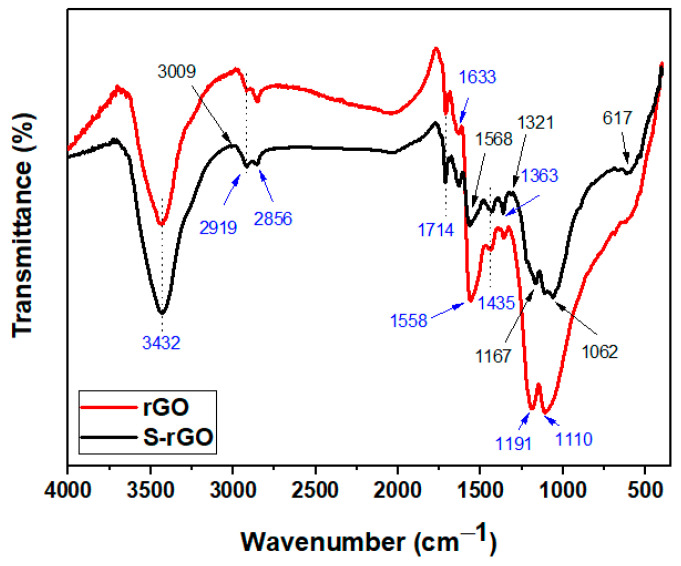
FTIR spectra of the reduced graphene oxide (rGO) and the silanated rGO (S-rGO).

Figure 4 shows the spectra of rGO and modified rGO (S-rGO). The spectra of rGO was typical and agreed with the literature [37], showing a broad peak at 3432 cm^−1^ due to the stretching vibrations of OH, NH and moisture and weak bands at 2919 and 2856 cm^−1^ for residual alkyl groups. The characteristic bands of carbonyl stretching and amine bending modes are assigned at 1714 and 1558 cm^−1^, respectively. After silanization, the appearance of new peaks at 1062 and 895 cm^−1^ assigned to Si–O–Si/Si–O–C indicates the successful silanization reaction [40]. Additionally, the slight shift in the peak corresponding to C=C (ca. from 1558 to 1568 cm^−1^) and the appearance of extra weak peaks at 1321 and 1167 cm^−1^ are due to the presence of silane moieties, providing more evidence for successful modification of rGO. The difficulty during the capture of γ-MPS-related peaks in the S-rGO indicates its relatively low incorporated quantity of γ-MPS, due to the deficiency in the available active sites for further modification on the rGO surface.

### 4.2. Rheological Properties

**Figure 5:**
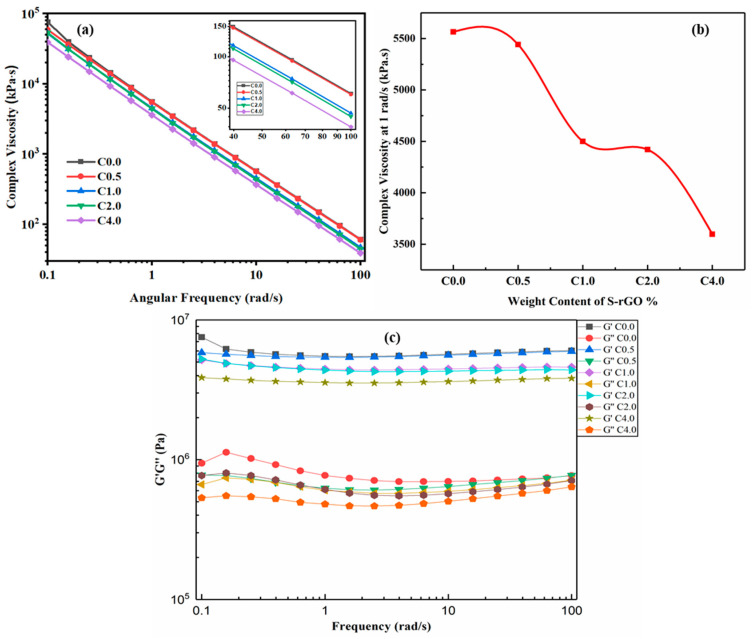
Rheological properties of the experimental composites (C0.0—C4.0) at 25 °C. (**a**) Complex viscosity vs angular frequency (Inset is a magnified view of the complex viscosity) (**b**) complex viscosity vs S-rGO wt.% at 1 rad/s (**c**).

Figure 5a–c shows the viscoelastic properties of all the uncured resin mixtures. Figure 5a depicts the complex viscosities versus frequency of the five experimental samples. For all the tested composites, the complex viscosity decreased as the frequency increased, representing strong non-Newtonian behavior (shear-thinning, that is, with increasing shear rate, the viscosity got reduced, which is termed as pseudo-plasticity). The complex viscosity values at 1 rad/s were selected and depicted in Figure 3b for comparison. It could be seen that the viscosity of C0.0 (the pristine resin) was the highest (5564.8 ± 36.23 kPa.s); however, with an increase in the S-rGO contents, the viscosity decreased to (5442.1 ± 38.43), (4499.3 ± 29.11), (4419.8 ± 39.52) and (3596.9 ± 31.17) kPa.s for C0.5, C1.0, C2.0 and C4.0 respectively. C4.0 has the lowest viscosity value (3596.9 ± 31.17 kPa.s). The decreased in viscosity can be observed with increasing S-rGO content. It revealed that the addition of a small amount of S-rGO altered the fluidity of the resin/filler suspension. However, no sign of a Newtonian plateau was perceived even at the lowest frequencies. The decrease in viscosity could be associated with the plasticizing behavior of S-rGO sheets, by enhancing polymer chains mobility to slide past each other. Consequently, the viscous nature of uncured resin/filler suspension gets decreased [41]. Figure 5c demonstrates the viscoelastic response of all the uncured resin mixtures. It can be inferred from Figure 5c that, the values of G” fluctuated with increasing frequency, however, G’ values remained stable even at a higher frequency. In all the cases, G’ is greater than G” at any frequency values which reflects that the mixture is stable even at a higher frequency. Since the values of G’ are larger than G,” this means that the elastic nature of the mixture is prevailing on the vicious nature. Therefore, it can be inferred that, although the addition of S-rGO reduced the viscosity of the suspension, it did not prevail on the elastic nature of it. 

### 4.3. Degree of Conversion

**Figure 6:**
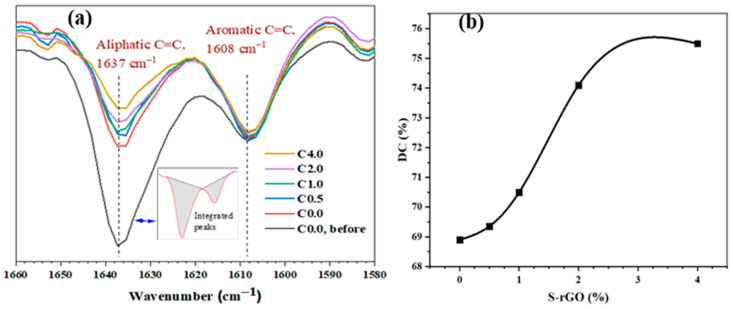
Representation of the double bond conversion for various SrGO-containing samples before (G0, before) and after curing (G0–G4) (**a**) characteristic FTIR spectra with peak area illustration (**b**) Obtained degree of conversion.

Figure 6a is an illustration of the FTIR peak (transmittance mode) of both the cured (C0.0–C4.0) and uncured (only C0.0 before curing is presented for simplicity) samples in the target wavenumbers between 1660 and 1580 cm^−1^. The intensities of the vinyl bands are reduced with increasing S-rGO in the specimen, which is proportional to the remaining mole fraction of aliphatic C=C bonds as well [10], indicating DC increase with S-rGO quantity increase. Figure 6b shows the value change of DC with respect to S-rGO quantity in the samples under investigation.

Figure 6a illustrates the FTIR spectra of the experimental model composites. It could be seen that the intensity of the vinyl double bonds (C=C, aliphatic, 1637 cm^−1^) descends as the concentration of the S-rGO increases in the composites; a spectrum of one uncured sample (C0.0) is shown as well for visibility. The DC, however, was calculated through comparison of the peak areas corresponding to the aliphatic and aromatic C=C bonds assigned at 1637 and 1608 cm^−1^, respectively, according to Equation (2); peak area integration was exemplified in the inset of Figure 6a. Figure 6b shows the relative DC for the investigated composites, revealing an increase in DC from (68.9 ± 0.65) % to (75.5 ± 0.71) % upon S-rGO amount increase from 0.0 to 4.0 wt.%. The curvature shape indicates a slow rate of DC increase at both low and high concentrations of S-rGO. However, a dramatic increase in DC was observed when the amount of S-rGO increased from 1.0 to 2.0 wt.% (C1.0 and C2.0), that is from DC (70.5 ± 0.62)–(74.1 ± 0.81)%.

This may be due to the low quantity of S-rGO at the beginning and the dispersion saturation effect at the end of the examined range. Graphitic materials are inactive and their inclusion, mostly in quantities as low as 10 wt.%, is rather physical in dental composites. The role of such materials is to enhance the existing properties of the composites. On one hand, graphene oxide may cause deactivation of the initiator system due to the inhibiting property of the aromatic hydroxyl of GO [42]. However, this effect may be limited after modification of GO [43]. Therefore, the observed increase in the viscosity of S-rGO containing composites could be attributed to the plasticizing effect. In this case, the graphitic sheets may facilitate mobility of the resins molecules, thus decreasing their viscosity even upon polymerization. On the molecular level, the interaction between the matrix components, dominated by hydrogen bonding, may be disturbed by the presence of carbon-based sheets. On the other hand, though the degree of conversion is increased due to composite viscosity enhancement as a result of the plasticizing effect of graphitic sheets, a negative effect on the depth of cure property is reported [44]. This effect, however, is dependent on the type of graphene and its sheet sizes.

### 4.4. Nano-Indentation Results

**Figure 7:**
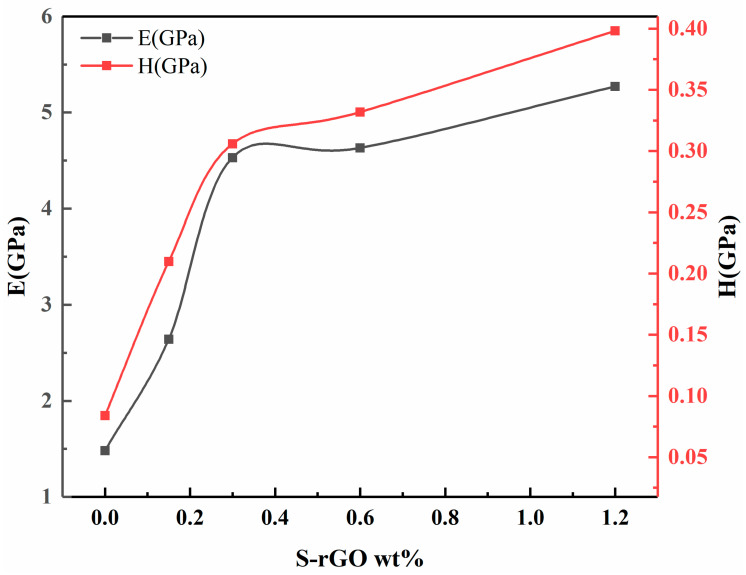
Nano hardness and elastic modulus of the base composite (C0.0) and S-rGO/SiO_2_ hybrid composites (C0.5–C4.0).

Figure 7 exhibits the average nano-hardness and elastic modulus values measured for cured pristine (C0.0) and S-rGO reinforced nanocomposites (C0.5, C1.0, C2.0, C4.0). The modulus and nano-hardness were is acquired using the previously mentioned Oliver–Pharr method [45] and the unloading curve, as given in the following Equations (3) and (4) [14,46].

(3) $$\frac{1}{E_{r}}=\frac{1-\nu^{2}}{E}+\frac{1-\nu_{i}{}^{2}}{E_{i}}\;$$

(4) $$E_{r}=\frac{\sqrt{\pi }}{2}\;\frac{dp}{dh}\;\frac{1}{\sqrt{A}},$$

where *E* is the modulus of the sample, ν is the Poisson’s ratio normal to loading of the sample, *E_i_* is the elastic modulus of the indenter, $\nu_{i}$ is the indenter Poisson’s ratio no., *dp*/*dh* is contact stiffness (S) in which p represents the unloading force and *h* is the corresponding indentation depth and *A* is the contact area. The indentation hardness is defined as in Equation (5):

(5) $$H=\;\frac{F_{max}}{A},$$

where *F_max_* is the maximum load normal to the surface and *A* is the indentation area at the maximum load.

From the nanomechanical response of all the test groups as given in Figure 7, it can be inferred that the base composite (C0.0) with no S-rGO, resulted in hardness of (0.084 ± 0.0068 GPa) and elastic modulus of (1.48 ± 0.32 GPa). However, the addition of small traces of S-rGO (0.5 and 1.0 wt.%) to the base nanocomposite significantly increased the hardness to (0.21 ± 0.0081 and 0.31 ± 0.0076) and elastic modulus to (2.64 ± 0.41 and 4.53 ± 0.33 GPa) respectively. Further increase in S-rGO content (2.0 and 4.0 wt.%), gradually increased hardness (0.33 ± 0.0077and 0.40 ± 0.0089) and elastic modulus (4.63 ± 0.42 and 5.27 ± 0.38) respectively. From previous studies, It is evident that the hardness and modulus increase significantly with the loading of hybrid nanoparticles (SiO_2_/Graphene) [27]. The increase in hardness can be attributed to the reduction in molecular chain mobility and resistance to indentation caused by the incorporated hybrid filler in the nanocomposites [47]. Thus, the resistance of the composite against the distortion by the applied force increased [48]. Moreover, it is expected that by increasing hybrid nanoparticles; (SiO_2_-S-rGO), the indenter interacts with more nanoparticles. The increase in the modulus may be attributed to the reduction in free volumes and enhanced cross-link density in the presence of hybrid nanoparticles. Well-dispersed nanoparticles tend to fill the cavities and interlink the matrix by bridging the chains to each other, resulting in a reduction in the total free volume and increase cross-link density of the cured composite. Consequently, the cured composite has restricted segmental chain mobility and enhanced stiffness [39,40]. The increase in hardness and modulus also corroborates the observed increase in DC. 

### 4.5. Morphology 

Usually, the performance of nanocomposites is greatly influenced by two key factors: (i) dispersion of the filler and (ii) interfacial interaction. Uniform dispersion of filler nanoparticles and strong interfacial interaction lead to magnificent mechanical properties of reinforced composites [49]. 

**Figure 8:**
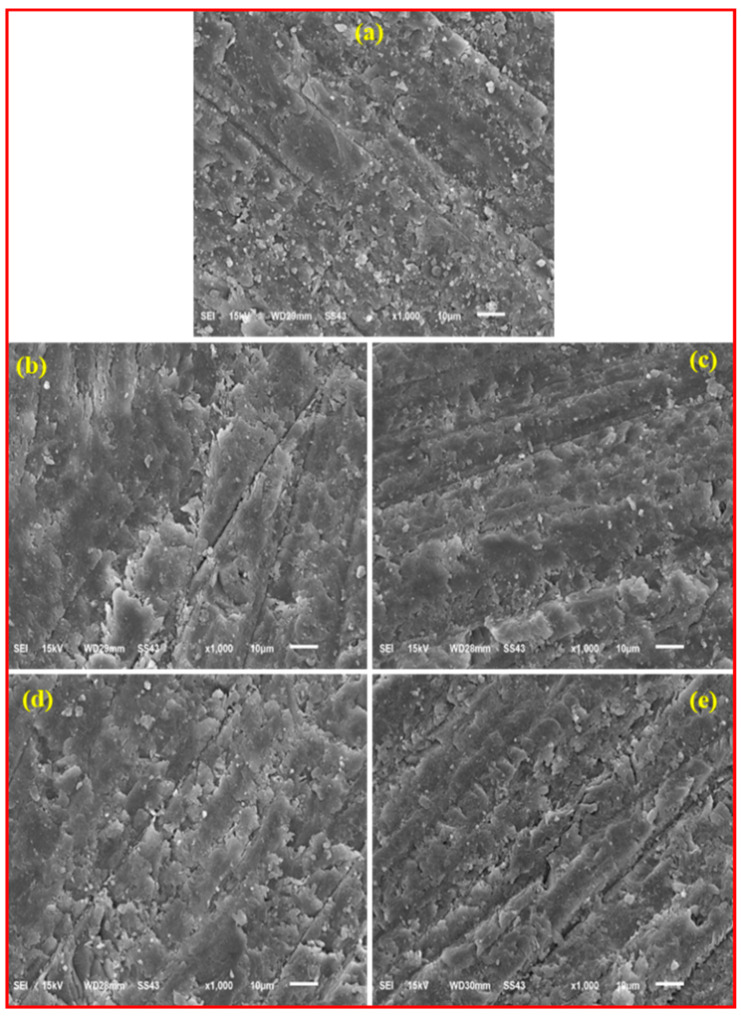
Scanning electron microscopy (SEM) images of fractured surface (**a**) Base composite with 30% SiO_2_ (C0.0), (**b**) (C0.5), (**c**) (C1.0), (**d**) C2.0, (**e**) C4.0.

To get an insight into the resin/filler interaction and the dispersion of silica and S-rGO hybrid fillers, we analyzed the fractured microstructure of all the test groups by SEM (Figure 8a–e). The SEM analysis revealed that, in all the experimental groups, SiO_2_ (C0.0) and hybrid SiO_2_/S-rGO (C0.5–C4.0) nanofillers are homogeneously distributed in the BisGMA/TEGDMA matrix. In the base composite (C0.0), the SiO_2_ nanoparticles are uniformly dispersed and embedded in the BisGMA/TEGDMA matrix. However, some deboned SiO_2_ particles could be observed on the surface which may imply week filler/matrix interaction (Figure 8a). On the other hand, composites reinforced with hybrid SiO_2_/S-rGO filler demonstrated a rough and wavy fracture surface, exhibiting deeper and irregular cracks appearing on the fracture surface. Moreover, no deboned SiO_2_ or S-rGO particles could be observed. This indicates that the SiO_2_-S-rGO surfaces are most possibly coated by BisGMA/TEGDMA resin. The appearance of micro indents and rough fractographs are initiated from the pulling-out of the resin coated SiO_2_-S-rGO. Therefore, it could be inferred that the presence of SiO_2_-S-rGo hybrid filler has impeded the propagation of cracks and demonstrated substantially toughening behavior. These investigations corroborate the improved mechanical properties of BiSGMA/TEGMDA composites with SiO_2_-S-rGO hybrid filler.

### 4.6. Surface Roughness

**Figure 9:**
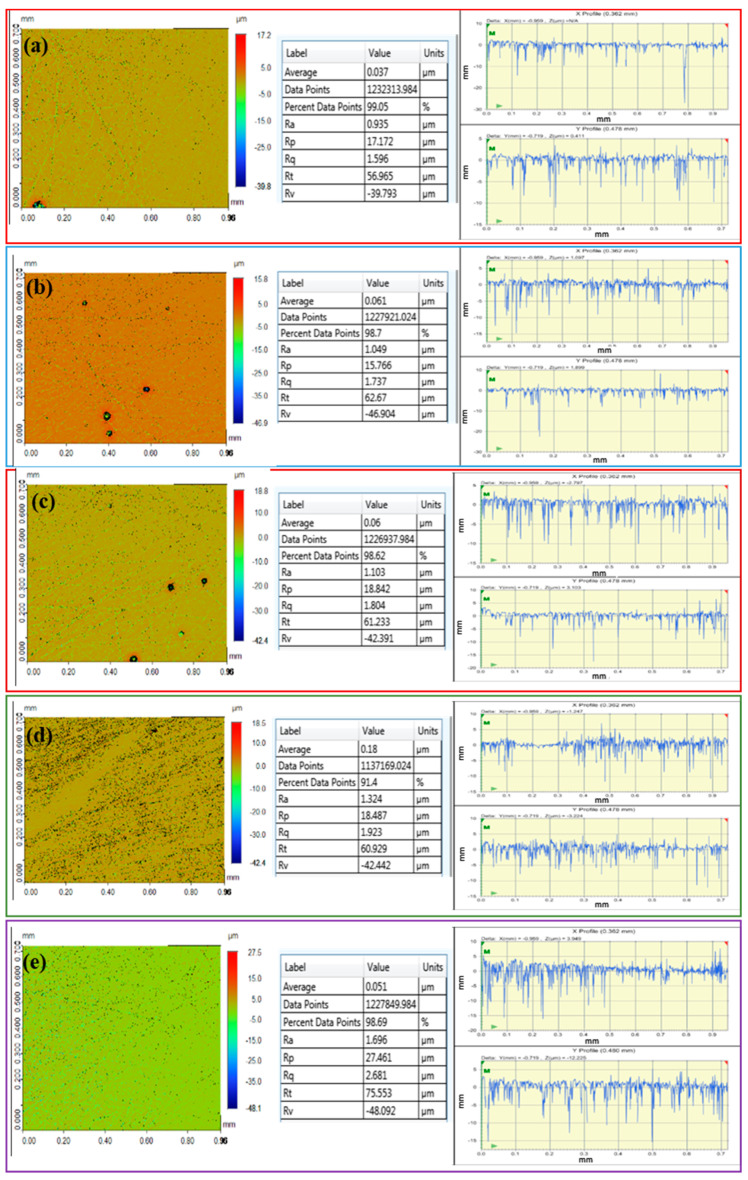
Topographic profilometry and surface roughness of the various resin composites with different geometrical topographies, (**a**) C0.0, (**b**) C0.5, (**c**) C1.0, (**d**) C2.0, (**e**) C4.0.

The topographic images and surface roughness of the five experimental resin composites with different geometrical topographies are shown in Figure 9. These topographic images can be used to measure the surface roughness by various mathematical approaches; however, the root mean squared roughness (RMS: Rq) and average roughness (Ra) are commonly used parameters for surface roughness [50,51,52,53,54,55]. The resulting Rq roughness values of test groups were measured as (1.60 ± 0.51 μm), (1.74 ± 0.84 μm), (1.80 ± 0.65 μm), (1.92 ± 0.71 μm) and (2.71 ± 0.81 μm) for C0.0, C0.5, C1.0, C2.0 and C4.0, respectively. Similarly, the obtained average surface roughness (Ra) values were recorded as (0.93 ± 0.36 μm), (1.05 ± 0.27 μm), (1.10 ± 0.33 μm), (1.32 ± 0.28) μm and (1.70 ± 0.29 μm) respectively. The surface roughness of composite materials is affected by a combination of many factors including intrinsic characteristics. The intrinsic characteristics of composites are associated with the composition (filler type, shape, size, distribution), type of resin matrix, degree conversion and the bond efficiency at the filler/matrix interface [52]. From our results, it can be observed that the surface roughness of the experimental composites slightly increases with increasing S-rGO traces. The gradual increase in surface roughness of experimental nanocomposites by incorporation of S-rGO (C0.5, C1.0, C2.0, C4.0) can be associated with the decoration of the modified SiO_2_ nanoparticles on S-rGO surface resulting in irregular surface [56], indicating induced crack deflection during fracture by S-rGO sheets [57]. Consequently, this may enhance not only the stiffness but also fracture toughness and ductility of the composite [58]. 

## 5. Conclusions 

BisGMA/TEGDMA nanocomposites filled with S-rGO/SiO_2_ hybrid filler were successfully fabricated by changing S-rGO concentration (0.0–4.0 wt.%) in combination with 30 wt.% silanized fumed SiO_2_. The study demonstrated that the addition of SiO_2_−S-rGO hybrid filler into BisGMA/TEGDMA resin system improves the rheological properties and significantly enhances the mechanical properties (nano-hardness, elastic modulus). Increasing S-rGO content in the suspension decreased the viscosity which subsequently resulted in higher DC and crosslink density of the composites. Thus the incorporation of S-rGO/SiO_2_ hybrid filler resulted in a compact and tough composite structure with improved nano-hardness and elastic modulus. Therefore, S-rGO in combination with surface modified fumed SiO_2_ could be a potential hybrid filler for dental composites. The concept of using S-rGO/SiO_2_ hybrid filler offers various prospects to alter the physical and mechanical properties of dental composites. Further modifications and optimization of this hybrid filler will unfold new ways to fabricate high-performance dental composites. Aesthetics and depth of cure are some of the concerned areas which could be explored. 

## Figures and Tables

**Table 1:** Composition of the experimental dental composites C0.0–C4.0.

Composite	Resin (wt.%)	Filler (wt.%)		Initiator System (wt.%)
		S-A200	S-rGO wt.% w.r.t S-A200	
C0.0	69	30	0	1
C0.5	69	30	0.5	1
C1.0	69	30	1.0	1
C2.0	69	30	2.0	1
C4.0	69	30	4.0	1

Resins: 1:1 (by weight) of BisGMA and TEGDMA. Initiator system: 1:4 (by weight) of CQ and DMAEMA.
